# Sialyltransferase Inhibitor Ac_5_3F_ax_Neu5Ac Reverts the Malignant Phenotype of Pancreatic Cancer Cells, and Reduces Tumor Volume and Favors T-Cell Infiltrates in Mice

**DOI:** 10.3390/cancers14246133

**Published:** 2022-12-12

**Authors:** Laura Miró, Júlia López, Pedro E. Guerrero, Neus Martínez-Bosch, Noemí Manero-Rupérez, Mireia Moreno, M. Rosa Ortiz, Esther Llop, Pilar Navarro, Rosa Peracaula

**Affiliations:** 1 Biochemistry and Molecular Biology Unit, Department of Biology, University of Girona, 17003 Girona, Spain; 2 Girona Biomedical Research Institute (IDIBGI), 17190 Girona, Spain; 3 Cancer Research Program, Hospital del Mar Medical Research Institute (IMIM), Unidad Asociada IIBB-CSIC, 08003 Barcelona, Spain; 4 Pathology Department, Josep Trueta University Hospital, 17007 Girona, Spain; 5 Institute of Biomedical Research of Barcelona (IIBB)-CSIC, 08036 Barcelona, Spain; 6 Institut d’Investigacions Biomèdiques August Pi i Sunyer (IDIBAPS), 08036 Barcelona, Spain

**Keywords:** pancreatic cancer, sialyltransferase inhibitor, sialic acid, sialyl-Lewis x, E-selectin adhesion, invasion, syngeneic mice, immune component

## Abstract

**Simple Summary:**

An increase of sialic acid in the cancer cells’ surface is a hallmark of tumours, including pancreatic cancer, and it has been related to tumour malignancy and immune suppression. In this work, we have assessed for the first time the potential of the sialyltransferase inhibitor, Ac_5_3F_ax_Neu5Ac, to reduce tumor sialylation in pancreatic cancer and to revert its malignant phenotype. We have shown that Ac_5_3F_ax_Neu5Ac treatment on human pancreatic cancer cells decreased their sialic acid, reduced their E-selectin adhesion—the prior step to tumour extravasation—and their migration and invasion capabilities. In addition, subcutaneous pancreatic tumours generated on immunocompetent mice that were treated with Ac_5_3F_ax_Neu5Ac showed a reduced growth and increased tumour infiltrating lymphocytes. These results show that the targeting of tumour sialoglycans in pancreatic cancer reverts its malignant phenotype and favours anti-tumour immune surveillance, which opens the way to use sialyltransferase inhibitors as a novel therapeutic strategy against this dismal disease.

**Abstract:**

Hypersialylation is a feature of pancreatic ductal adenocarcinoma (PDA) and it has been related to tumor malignancy and immune suppression. In this work, we have evaluated the potential of the sialyltransferase inhibitor, Ac_5_3F_ax_Neu5Ac, to decrease tumor sialoglycans in PDA and to revert its malignant phenotype. Sialoglycans on PDA cells were evaluated by flow cytometry, and the functional impact of Ac_5_3F_ax_Neu5Ac was assessed using E-selectin adhesion, migration, and invasion assays. PDA tumors were generated in syngeneic mice from KC cells and treated with Ac_5_3F_ax_Neu5Ac to evaluate tumor growth, mice survival, and its impact on blocking sialic acid (SA) and on the tumor immune component. Ac_5_3F_ax_Neu5Ac treatment on human PDA cells decreased α2,3-SA and sialyl-Lewis^x^, which resulted in a reduction in their E-selectin adhesion, and in their migratory and invasive capabilities. Subcutaneous murine tumors treated with Ac_5_3F_ax_Neu5Ac reduced their volume, their SA expression, and modified their immune component, with an increase in CD8^+^ T-lymphocytes and NK cells. In conclusion, Ac_5_3F_ax_Neu5Ac treatment weakened PDA cells’ malignant phenotype, thereby reducing tumor growth while favoring anti-tumor immune surveillance. Altogether, these results show the positive impact of reducing SA expression by inhibiting cell sialyltransferases and open the way to use sialyltransferase inhibitors to target this dismal disease.

## 1. Introduction

Pancreatic ductal adenocarcinoma (PDA) is the fourth leading cause of cancer deaths in western countries with a 5-year survival of 11% [1,2]. This extremely poor outcome is mainly due to its aggressiveness and delay in diagnosis, when metastasis is already present [3,4]. The current standard of care for PDA patients includes chemotherapeutic cocktails that are highly toxic with limited specificity [4], still leaving the majority of PDA patients to die of metastatic disease, which highlights the urgent need to develop novel therapeutics that target not just the primary tumor, but also the biological vulnerabilities of metastatic PDA cells. 

Immunotherapy has offered encouraging results in several solid tumors, albeit as yet these approaches in PDA have failed to show clear benefits in clinical trials and remain to have proven value [5]; however, there is strong evidence that immune infiltrates play an important role in shaping PDA microenvironment and the course of the disease [6].

Tumors show an aberrant glycosylation pattern. In PDA, the aberrant glycosylation profile, which includes an increase in sialyl-Lewis x (sLe^x^), in truncated *O*-glycans (Tn and sTn), and in branched and fucosylated glycans, has been associated with disease progression and poor prognosis. Tumor sialoglycans facilitate cancer cell migration and metastasis and promote tumor immune evasion, emerging as potent immunomodulators [7,8].

Hypersialylation is therefore being considered as a target for cancer immunotherapy and, to this end, several sialyltransferase (ST) inhibitors have been developed [9,10]. The glycomimetic cell-permeable ST inhibitor Ac_5_3F_ax_Neu5Ac, which acts as a competitive ST inhibitor [10], has been shown to decrease tumor cell sialoglycans, the adhesive and migratory capability of melanoma cells, while it favors the anti-tumor immune response [11,12].

In PDA, the increase of sialylation through the overexpression of the α2,3-STs ST3GAL3 and ST3GAL4 increased sLe^x^ expression and facilitated the invasion and metastasis processes in PDA cellular and animal models [13,14]. The importance of sialoglycans in PDA was confirmed when the decrease of PDA sialylation, in particular of sLe^x/a^ tumor antigens, achieved by the knockdown (KD) of ST3GAL3 and ST3GAL4 impaired PDA cell migration, invasion, and E-selectin-dependent adhesion in vitro [15].

Blocking sialylation by modifying ST expression or by sialidase treatment has been used to decipher the role of tumor sialic acids, but the application of these approaches to the clinical setting is complex. Nonetheless, the recent discovery of ST inhibitors have represented a new strategy to pharmacologically inhibit sialic acid (SA) expression in tumors and could ease the path for a putative translation to the clinic. The inhibition of tumor cell sialoglycans in PDA by specific ST inhibitors is expected to revert the E-selectin adhesion and invasion capabilities and to enhance the immune response against PDA. To address these issues, in this work we have assessed the impact of the ST inhibitor, Ac_5_3F_ax_Neu5Ac, to block aberrant tumor sialoglycans such as sLe^x^ in PDA cell models and to counteract tumor migration and invasion—prior steps to metastasis formation. Furthermore, we have evaluated how the blocking of the sialoglycans of PDA tumors generated in syngeneic mice has an impact on reducing tumor growth and favors the anti-tumor immune response.

## 2. Materials and Methods

### 2.1. Reagents and Cell Lines

Human PDA cell lines BxPC-3 and Panc-1 were obtained from the cancer cell repository at IMIM (Barcelona, Spain) and Capan-1 was from the ATCC (Manassas, VA, USA). Murine PDA cell lines Ptf1-KRAS^G12D^p53^+/+^ (KC) and Pdx1-KRAS^G12D^p53^+/−^ (KPC) were a kind gift of Dr. C. Guerra (CNIO, Madrid, Spain). Cells were routinely cultured as described [15]. Ac_5_3F_ax_Neu5Ac (Merck, Darmstadt, Germany) was reconstituted in DMSO to a 90.66 mM stock solution and diluted with complete media. For untreated cells, complete media with the corresponding concentration of the vehicle (DMSO) was used. Briefly, cells at 75% of confluence were trypsinized and seeded for 24 h, then Ac_5_3F_ax_Neu5Ac or the vehicle was added to the treated or untreated cells, respectively, during 72 h until they were trypsinized for further assays. The effects of Ac_5_3F_ax_Neu5Ac treatment on cell proliferation was assessed with FC by counting cell numbers of treated and untreated cells and viability after staining the cells with Sytox Blue. Antibodies, lectins, and secondary reagents used in flow cytometry (FC), Western Blot (WB), and immunohistochemistry (IHC) are listed in Table 1. 

### 2.2. Flow Cytometry

Detection of cell SA determinants was performed by indirect fluorescence, as previously described [15]. The cells’ median fluorescence normalized vs. the corresponding negative control was determined using an Acea NovoCyte^®^ Flow Cytometer (Agilent, Santa Clara, CA, USA) with the NovoExpress^®^ software. Cellular suspension from the murine tumors was prepared as described [16] and kept at −80 °C in FBS and 10% DMSO until used. For the immune component and tumor infiltrates analysis, samples were processed using the Cytek^®^ Aurora spectral flow cytometer (Cytek Biosciences, Fremont, CA, USA). Briefly, cells were defrosted, resuspended in PBS and 5% FBS, and then incubated with Live/Dead staining (Life Technologies, Carlsbad, CA, USA). After washings, cells were blocked with CD16 antibody for 15 min at 4 °C, washed again, and then incubated with a panel of fluorophore-conjugated antibodies (Table 1) for 1 h at 4 °C. At least 1.5 × 10^5^ live cells were gated and analyzed using the SpectroFlo software.

### 2.3. Protein Lysates and Western Blot (WB)

Protein lysates were obtained in RIPA buffer, quantified, and 50 µg of total protein were used for WB analyses as previously described [15].

### 2.4. E-Selectin Binding Assay

Cell adhesion to recombinant human E-selectin (rhE-selectin) was performed with minor modifications, as previously described [15], using 2 µg/mL of rhE-selectin/Fc Chimera (R&D Systems, Minneapolis, MN, USA), which was incubated to a Fc antibody (Millipore, Darmstadt, Germany) and was previously bound to a 96-well maxisorp microplate (Thermo Fisher Scientific, Waltham, MA, USA). A total of 75,000 cells/well for BxPC-3 and 50,000 cells/well for Capan-1, treated and non-treated with Ac_5_3F_ax_Neu5Ac, were seeded by quintuplicate into the rhE-selectin coated plates and incubated for 1 h at 37 °C. After washings, the remaining rhE-selectin-bound cells were estimated with the cell proliferation assay MTS (Promega, Madison, WI, USA), following the manufacturer’s protocol. The mean absorbance of the adhered cells was normalized by the corresponding negative control (wells without rhE-selectin). Cell adhesion was expressed by dividing the mean absorbance of treated cells by the mean of the untreated cells.

### 2.5. Transwell Migration and Invasion Assays

Cell migration and invasion were evaluated using modified Boyden chambers in 24-well plates, as previously described [15]. For migration, 2.5 × 10^4^ cells for BxPC-3, 5 × 104 for Capan-1, 2.5 × 10^4^ for Panc-1, or 3.5 × 10^4^ for KC were seeded in the top chamber of inserts; for invasion, 4 × 10^4^ cells for BxPC-3, 5 × 10^4^ for Capan-1, 4 × 10^4^ for Panc-1, or 2.5 × 10^4^ for KC were used. Then, 500 µL of complete media (10% FBS for BxPC-3, Panc-1 and KC, and 20% FBS for Capan-1) was added at the bottom as a chemoattractant. Cells were left to migrate for 18 h for BxPC-3, 22 h for Capan-1, or 24 h for Panc-1 or KC, and allowed to invade for 24 h for BxPC-3, 48 h for Panc-1 and KC, or 72 h for Capan-1. 

### 2.6. Syngeneic Mice Tumor Generation

A total of 20 adult C57BL female mice were acquired from Charles River and were housed in the animal facility of the Barcelona Biomedical Research Park (PRBB). For tumor generation, 0.5 × 10^6^ KC cells were resuspended in DMEM and Matrigel was added in 1:1 ratio. The cells were injected subcutaneously at the mice posteriors flanks. After two weeks approximately, when palpable tumors of around 100 mm^3^ appeared, mice were grouped into four groups (a–d) and treated with (a) 10 mg/kg Ac_5_3F_ax_Neu5Ac (N = 7), (b) 20 mg/kg Ac_5_3F_ax_Neu5Ac (N = 5), (c) vehicle (DMSO) (N = 3), and (d) PBS (N = 3). The two last-mentioned groups (c and d) were considered as the control group. Intra-tumoral injections were administered thrice per week under isoflurane anaesthesia during 15 days or until the tumor volume exceeded 1000 mm^3^. Mice were then sacrificed in CO_2_ euthanasia chamber and tumor samples were collected for posterior analyses. Mice presenting compromised general welfare during the experiment were sacrificed following ethical guidelines from the European Animal Research Association. Tumors were weighted and the three perpendicular diameters were measured with a vernier caliper to estimate the tumor volume. For histology and IHC, tumors were fixed in buffered formalin for 24 h, dehydrated, and embedded in paraffin.

### 2.7. Immunohistochemistry

IHC was performed as previously described [17]. The histological analysis and scoring of the tissues were performed in 10 fields at 20× per tissue by pathologists from the Hospital Dr. Josep Trueta of Girona.

### 2.8. Statistical Analyses

All statistical analyses and the corresponding figures were performed using GraphPad-Prism8. Normality analyses were performed using the Shapiro–Wilk normality test. Statistical differences between groups were assessed using unpaired Student’s *t*-test for parametric comparisons. Statistically significant results were considered when *p* < 0.05 using a 95% confidence interval. All data are presented as mean ± SD of at least three independent experiments. Kaplan–Meier survival test and long-rank test were used to plot mice survival. 

## 3. Results

### 3.1. Ac_5_3F_ax_Neu5Ac Reduced Cell Surface Sialylation in BxPC-3, Capan-1 and Panc-1 Cells

**Figure 1:**
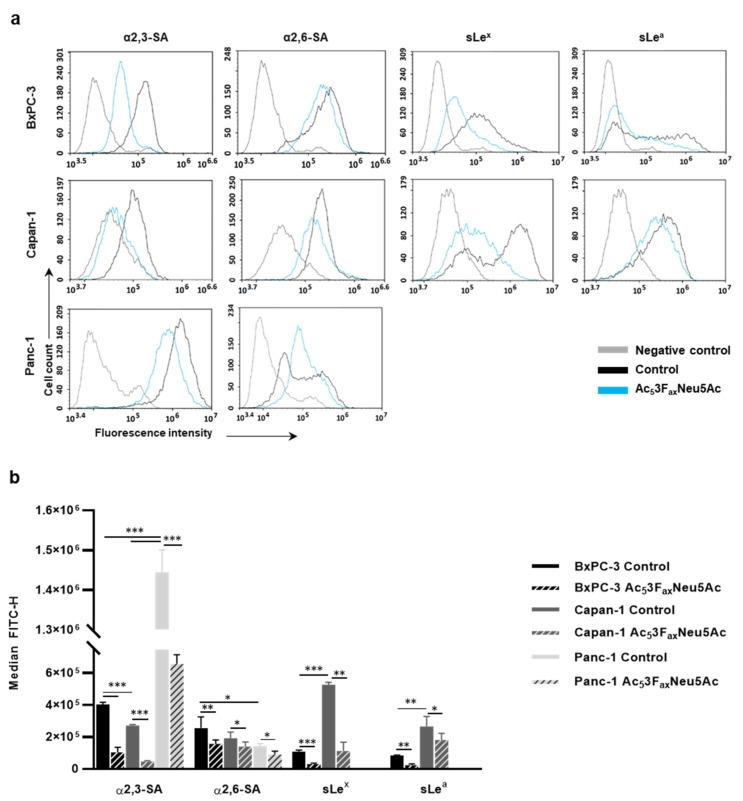
Analysis of Ac_5_3F_ax_Neu5Ac treatment on cell sialylation in BxPC-3, Capan-1, and Panc-1 cells. (**a**) Representative histograms of the sialylated glycans expression on the cell surface of treated and control BxPC-3 (top panel), Capan-1 (medium panel), and Panc-1 (bottom panel) cells α2,3-SA (detected by MAA-II lectin) α2,6-SA (detected by SNA lectin), sLe^x^ (mAb CSLEX), and sLe^a^ (mAb 121SLE) (from left to right). Description of what is contained in the first panel; (**b**) Median fluorescence for each glycan structure of treated and control BxPC-3, Capan-1, and Panc-1 cells are represented. Plots show the mean ± SD of at least three independent experiments. * *p* < 0.05, ** *p* < 0.01 and *** *p* < 0.001.

Before investigating the effect of the ST inhibitor Ac_5_3F_ax_Neu5Ac to decrease cell sialic acid (SA), the expression levels of sLe^x^, sLe^a^, α2,3-SA, and α2,6-SA were characterized by FC in three human PDA cell lines: BxPC-3, Capan-1, and Panc-1 (Figure 1), which represent the different degrees of tumor cell differentiation and of PDA genetic complexity. All cell lines expressed similar levels of α2,6-SA, while for α2,3-SA, Panc-1 was the highest expressing cell line, followed by BxPC-3, and showed a 5.3-fold increase and 1.5-fold increase, respectively, vs. Capan-1 (Figure 1). Interestingly, all cell lines expressed much higher α2,3-SA levels than α2,6-SA, which are in accordance with the pattern of expression of α2,3/6-SA of other PDA cell lines reported in the literature [15,18]. Regarding sialyl-Lewis antigen, Capan-1 expressed nearly a 5-fold higher level of sLe^x^ and 3-fold higher level for sLe^a^ than BxPC-3 cells, while Panc-1 did not express any of these sialyl-Lewis antigens (Figure 1).

A dose-finding study showed that Ac_5_3F_ax_Neu5Ac acted in a dose-dependent manner, and for BxPC-3 and Panc-1, the minimum dose that allowed the maximum reduction in SA expression was at 100 µM at 72 h, while for Capan-1 cells it was at 400 µM at 72 h. The used concentrations of Ac_5_3F_ax_Neu5Ac did not alter tumor cell morphology when assessed by microscopy; moreover, no significant changes in the number of cells and their viability were detected in any of the cell lines by FC, which is similar to described reports with other tumor cell lines [11,19].

In BxPC-3, Ac_5_3F_ax_Neu5Ac treatment led to a reduction of up to 74% for α2,3-SA, 32% for α2,6-SA, 85% for sLe^x^, and 76% for sLe^a^ (Figure 1). For Capan-1, Ac_5_3F_ax_Neu5Ac treatment caused a reduction of 83% for α2,3-SA, 28% for α2,6-SA, 80% for sLe^x^, and 37% for sLe^a^; and for Panc-1, the reductions were 55% for α2,3-SA and 38% for α2,6-SA (Figure 1). 

Moreover, in order to assess the changes in the sialylation pattern of cell glycoproteins of BxPC-3, Capan-1, and Panc-1 after Ac_5_3F_ax_Neu5Ac treatment, we analyzed the changes in expression of α2,3-SA, α2,6-SA, and sLe^x^ from cell lysates by WB (Supplementary Figure S1 and File S1). The inhibitor caused a notable reduction in α2,3-SA in all cell lines, being higher in Capan-1 and BxPC-3 compared to Panc-1, and significantly reduced sLe^x^ expression in both Capan-1 and BxPC-3, which is in line with FC results. The expression of α2,6-SA did not show a specific staining in the cell lines, and the treatment with the inhibitor did not reveal any significant change in α2,6-SA staining. 

### 3.2. Ac_5_3F_ax_Neu5Ac Impaired E-Selectin Binding in BxPC-3 and Capan-1 Cells

**Figure 2:**
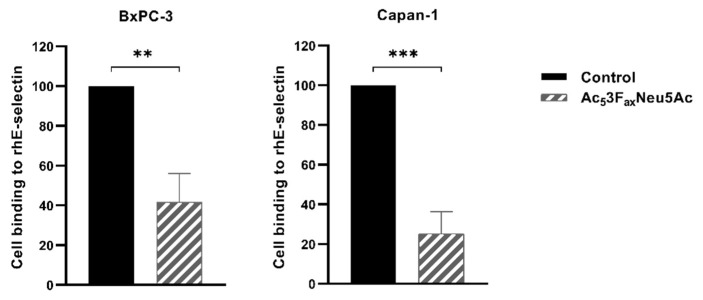
Ac_5_3F_ax_Neu5Ac treatment impaired cell adhesion to rhE-selectin in BxPC-3 and Capan-1 cells. BxPC-3 (**left**) and Capan-1 (**right**) cell adhesion was quantified after 1 h 30 min incubation over rh-E-selectin-Fc Chimera coated 96-well plates (previously treated with Fc secondary antibody) with MTS-based colorimetric assay. Plots represent mean ± SD of adherent cells normalized to non-treated (control) cells of at least three independent experiments. Unpaired Student *t*-test was performed ** *p* < 0.01 and *** *p* < 0.001.

To explore if the high decrease of sLe^x/a^ levels, which are E-selectin ligands involved in cancer cell metastasis, in BxPC-3 and Capan-1 Ac_5_3F_ax_Neu5Ac-treated cells can lead to alterations in their E-selectin adhesion, we first evaluated their ability to adhere to rhE-selectin. Capan-1 showed slightly higher adhesion to E-selectin than BxPC-3, a difference that might be explained by the higher sLe^x^ and sLe^a^ levels of Capan-1 compared to BxPC-3. Cells treated with BxPC-3 and Capan-1 significantly reduced their adhesion capacity to rhE-selectin, by 58% and 75%, respectively (Figure 2), which is in accordance with the decrease in their sLe antigen levels.

### 3.3. Ac_5_3F_ax_Neu5Ac Reduced PDA Cells Migration and Invasion

**Figure 3:**
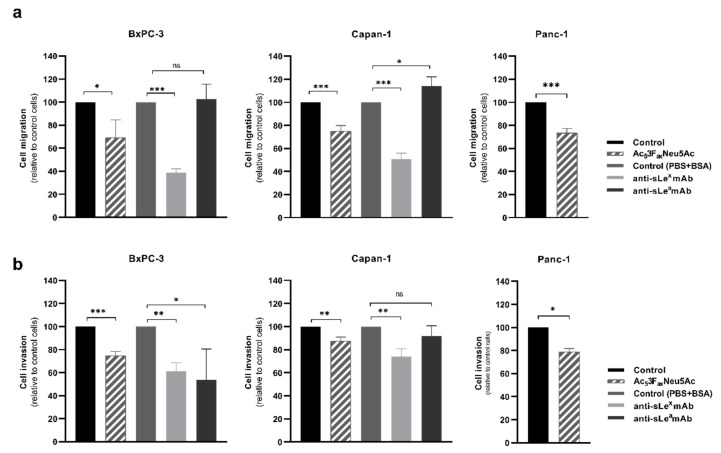
Ac_5_3F_ax_Neu5Ac treatment impaired cell migration (top) and invasion (bottom) of BxPC-3, Capan-1, and Panc-1 cells. BxPC-3 (left), Capan-1 (medium), and Panc-1 (right) cells were pre-treated for 72 h with ST inhibitor and allowed to (**a**) migrate through Collagen type-I coated transwells for 18 h for BxPC-3, 22 h for Capan-1, and 24 h for Panc-1 cells, or (**b**) invade in Matrigel-coated inserts for 24 h, 72 h, or 48 h for BxPC-3, Capan-1, and Panc-1, respectively. Migrated or invading cells were fixed, stained, and the area occupied by the cells was quantified with ImageJ software. Plots represent mean ± SD of migrated/invaded cells normalized to non-treated (control) cells from at least three independent experiments, ns: no significance. * *p* < 0.05; **, *p* < 0.01 and ***, *p* < 0.001.

To investigate the potential of Ac_5_3F_ax_Neu5Ac to modulate the migratory and invasive capacity of BxPC-3, Capan-1, and Panc-1, cells were pre-treated with Ac_5_3F_ax_Neu5Ac and allowed to migrate/invade in modified Boyden chambers coated with Type-I collagen or Matrigel, respectively. Ac_5_3F_ax_Neu5Ac treatment significantly impaired cancer cell migration in all cell lines—by 30% in BxPC-3, 25% in Capan-1, and 27% in Panc-1 cells (Figure 3a)—and it also significantly reduced cell invasion—by 25% in BxPC-3, 13% in Capan-1, and 19% in Panc-1—in comparison to their respective untreated controls (Figure 3b). 

The level of reduction in migration was similar between the cell lines, although their pattern of expression of sialylated antigens was quite different among them. In order to determine if the decrease in migration observed in the treated cells could be in part explained by sLe^x^ and sLe^a^ reduced expression, Capan-1 and BxPC-3 cells, which express sLe antigens, were incubated with blocking mAbs against sLe^x^ and sLe^a^ for 20 min prior to their seeding into the transwells. The incubation with the anti-sLe^x^ mAb showed a reduction of 61% in BxPC-3 and of 49% in Capan-1 cells compared to their respective controls (Figure 3a). These levels of reduction were higher than the ones obtained after the Ac_5_3F_ax_Neu5Ac treatment, which could be explained by the non-complete blocking of sLe^x^ in the Ac_5_3F_ax_Neu5Ac-treated cells. Cells treated with anti-sLe^a^ mAb presented a similar migration capacity (BxPC-3) or a slight increase in migration, up to 14% (Capan-1), suggesting that sLe^a^ is not playing a role in modulating cell migration in these PDA cells. 

To determine the involvement of sLe antigens in the invasion of Capan-1 and BxPC-3, cells were also pre-incubated with the blocking mAbs against sLe^x^ and sLe^a^. The invasion capacity of BxPC-3 incubated with anti-sLe^x^ and anti-sLe^a^ was significantly reduced to 38% and 46%, respectively (Figure 3b). In Capan-1 cells, the incubation with anti-sLe^x^ caused a decrease in their invasive capacity to 26%, while invasion was not impaired by the anti-sLe^a^ antibody (Figure 3b). These results point out a relationship between the reduction of sLe^x^ expression caused by Ac_5_3F_ax_Neu5Ac treatment and the decrease in the invasiveness of PDA cells.

### 3.4. Ac_5_3F_ax_Neu5Ac Decreased SA Expression in KPC and KC Murine PDA Cell Lines, and Impaired KC Cells Migration and Invasion

**Figure 4:**
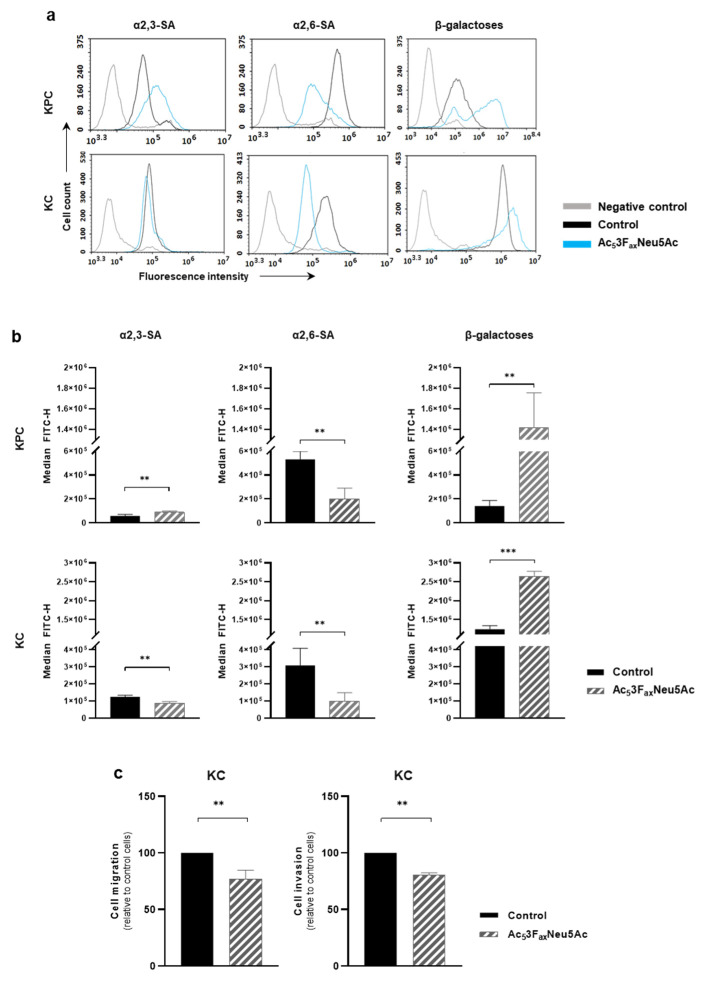
Ac_5_3F_ax_Neu5Ac reduced sialylation in KPC and KC murine pancreatic cancer cell lines and impaired cell migration and invasion capacity in KC cells. (**a**) Representative histograms showing the expression levels of α2,3-SA, detected by MAA-II lectin; α2,6-SA, detected by SNA lectin; or terminal β-galactose, detected by PNA lectin; (**b**) Median fluorescence for each glycan structure of treated and control KPC and KC cells are represented; (**c**) KC cells were pre-treated for 72 h with the ST inhibitor and allowed to (**left**) migrate through Collagen type-I coated transwells, for 24 h or (**right**) invade into Matrigel-coated inserts for 48 h. Migrated/invading cells were fixed, stained, and the area occupied by the cells was quantified with ImageJ software. Plots show mean ± SD of at least three independent experiments. ** *p* < 0.01 and *** *p* < 0.001.

To study the potential effect of Ac_5_3F_ax_Neu5Ac on pancreatic tumors in vivo, first, the sialoglycan expression pattern of murine PDA cell lines was derived from KC and KPC models, and the effect of the ST inhibitor on those cell lines was characterized. KPC and KC cell lines expressed high levels of α2,6-SA and moderate levels of α2,3-SA, while sLe^x^ and sLe^a^ antigens were not detected in any of them. The optimal dose of Ac_5_3F_ax_Neu5Ac and the best treatment time to decrease cell surface SA levels for KPC and KC was 200 µM at 72 h. Ac_5_3F_ax_Neu5Ac treatment led to a 63% decrease in α2,6-SA in KPC and 68% in KC cells (Figure 4a,b), while it caused a decrease of 30% in α2,3-SA in KC, whereas for KPC, Ac_5_3F_ax_Neu5Ac treatment resulted in an unexpected increase in α2,3-SA (Figure 4a,b). We also analyzed the concomitant augmentation of terminal galactose residues (detected by PNA lectin) due to the reduction of SA, and, accordingly, a large increase of galactose residues was detected in both treated cell lines (989.2% in KPC and 108.8% in KC) (Figure 4a,b). We selected the KC cell line to perform the following functional assays because it was the cell line that showed a decrease in both α2,3- and α2,6-SA upon treatment with Ac_5_3F_ax_Neu5Ac. In vitro migration and invasion assays showed that Ac_5_3F_ax_Neu5Ac treatment significantly impaired KC cells migration by 23% and their invasion by 20% vs. untreated cells (Figure 4c). 

### 3.5. Ac_5_3F_ax_Neu5Ac Treatment Reduced the Growth of the Tumors Generated by Subcutaneous Injection of KC Cells in Syngeneic Mice

**Figure 5:**
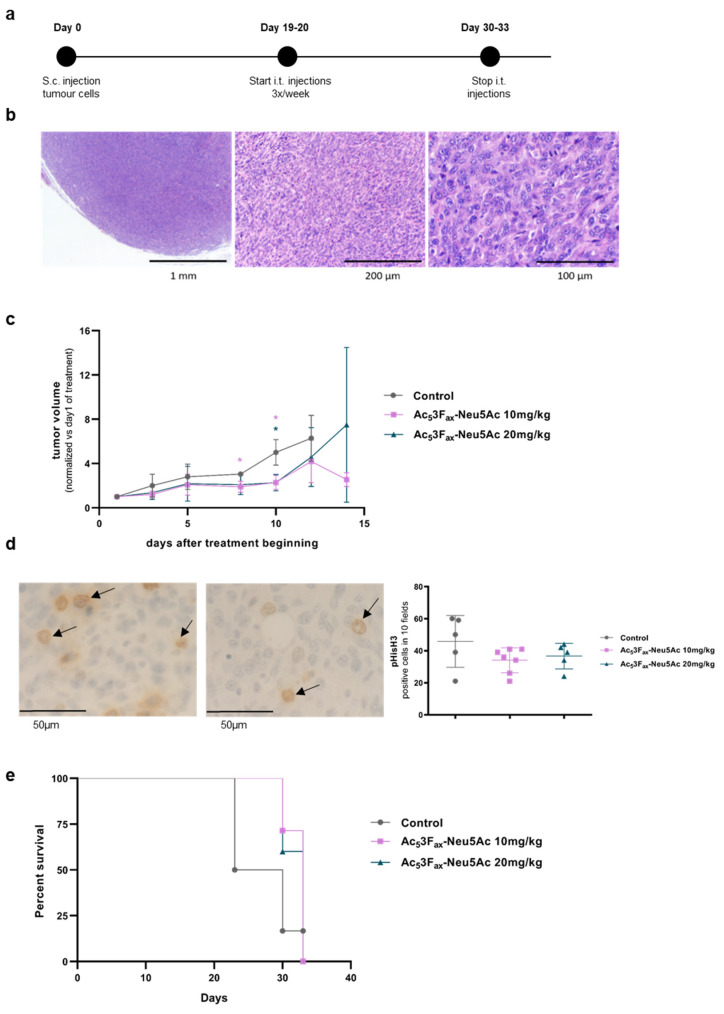
Effect of intratumoral Ac_5_3F_ax_Neu5Ac injections in tumor growth. KC cells were subcutaneously inoculated and tumors were treated with PBS, DMSO, or 10 mg/kg or 20 mg/kg Ac_5_3F_ax_Neu5Ac. (**a**) Time-based diagram of the experiment; (**b**) Haematoxylin-eosin staining of a representative tumor at different magnification (20×, 63× and 200×); (**c**) Tumor volume was measured thrice per week by measuring the three perpendicular diameters with a vernier caliper, since the beginning of the tumor treatment. Tumor volumes are represented as mean values of each group ± SD, and normalized vs. their tumor volume at the first day of administration. * *p* < 0.01.; (**d**) IHC with anti- pHisH3 of a representative PBS-treated control tumor (left) and 10 mg/kg Ac_5_3F_ax_Neu5Ac-treated tumor (right) at 400× (arrows indicate stained cells), and graph of the quantification of pHisH3 cell staining of tumors (**e**) Kaplan–Meier curve corresponding to mice survival of each mice group are represented.

To analyze the effect of Ac_5_3F_ax_Neu5Ac on tumor growth and its putative correlation to increased mice survival, KC cells were injected into C57BL syngeneic mice to generate subcutaneous tumors. Based on the data published by Büll et al. 2018 [12] and our own results, which indicate that cells recover basal sialic acid levels for 2 days (for α2,6-linked sialic acids) or 4 days (for α2,3-linked sialic acid) after Ac_5_3F_ax_Neu5Ac treatment removal, and considering the treatment schedule and dosage used in their in vivo study, we decided to apply the following treatment regimen: when palpable tumors of around 100 mm^3^ appeared, mice were intratumorally administered with 10 mg/kg or 20 mg/kg Ac_5_3F_ax_Neu5Ac for two weeks (Figure 5a). Injections with PBS or vehicle (DMSO) were used as controls. 

The subcutaneous tumors obtained were undifferentiated solid tumors, without any glandular structures, and with a low degree of immune components (Figure 5b). Some necrotic areas were detected (ranging from 5–70%), which tended to be higher in the tumors of both ST-inhibitor-treated groups (44.6% ± 20) compared with the control groups (31% ± 16.4). 

Intratumoral injections with 10 mg/kg of Ac_5_3F_ax_Neu5Ac resulted in a significant reduction of the tumor volume compared to the group of mice injected with PBS or DMSO (united as control group) eight days after the start of the treatment and were sustained until the end of the treatment (Figure 5c). Likewise, 20 mg/kg of Ac_5_3F_ax_Neu5Ac injections resulted in a decrease in tumor volume, although it was only statistically significant at day 10 of drug administration. At day 14, no control mice were alive, and the remaining three mice of the 10 mg/kg group kept their reduced tumor volume as in day 10, while the two remaining mice of the 20 mg/kg group showed a large dispersion in their tumor volume (Figure 5c). IHC showed higher phospho-histone H3 expression, which is a mitotic marker, in most tumors from the control groups vs. the treated tumors (Figure 5d), thereby supporting that tumor cell proliferation decreased by the treatment with Ac_5_3F_ax_Neu5Ac.

Regarding mice survival, the results showed a trend of 10 or 20 mg/kg of Ac_5_3F_ax_Neu5Ac administration to increase median mice survival time, although it was not statistically significant (Figure 5e). 

### 3.6. Ac_5_3F_ax_Neu5Ac Treatment Reduced SA Expression on Tumor Cells and Altered the Tumor Immune Component

**Figure 6:**
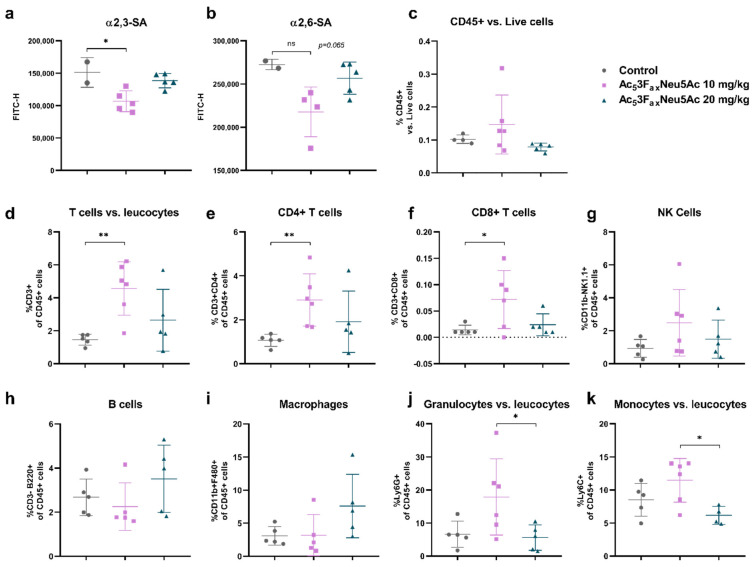
Intratumoral Ac_5_3F_ax_Neu5Ac injections reduced SA expression of tumor cells and altered the immune cell composition of the tumor. Analysis of cell surface sialoglycans expressed by tumor cells isolated from control, 10 mg/kg and 20 mg/kg Ac_5_3F_ax_Neu5Ac-treated tumors by flow cytometry. Dot plots showing mean ± SD of (**a**) α2,6-SA and (**b**) α2,3-SA levels of the different treatment groups (*n* = 2 for control; *n* = 6 for 10 mg/kg and *n* = 5 for 20 mg/kg Ac_5_3F_ax_Neu5Ac). (**c**–**j**) Analysis of immune cells of the different treatment groups (*n* = 5 for control; *n* = 6 for 10 mg/kg and *n* = 5 for 20 mg/kg Ac_5_3F_ax_Neu5Ac), dot plots showing mean ± SD of (**c**) CD45^+^ immune infiltrates, (**d**) CD3^+^ T cells, (**e**) CD4^+^ T cells, (**f**) CD8^+^ T cells, (**g**) CD11b^+^ NK1.1^+^ cells, (**h**) CD3-B220^+^ B cells, (**i**) CD11b^+^ F480^+^ macrophages, and (**j**) Ly6G^+^ or (**k**) Ly6C^+^ myeloid cells in the tumors of the different treatment groups. ns: no significance. * *p* < 0.05 and ** *p* < 0.01.

Tumors were collected after treatments and analyzed by FC. The tumors of the 10 mg/kg Ac_5_3F_ax_Neu5Ac group showed a reduction of 16% on α2,6-SA expression and a significant reduction of 24% on α2,3-SA expression in comparison to control mice tumor cells (Figure 6a,b). For tumors administered with 20 mg/kg of Ac_5_3F_ax_Neu5Ac, we could only detect a slight decrease in SA expression. 

Leukocyte marker CD45.2 was used to differentiate tumor cells (CD45.2 negative) and the immune cells (CD45.2 positive). An increase in the immune cell to tumor cell ratio was observed in the 10 mg/kg Ac_5_3F_ax_Neu5Ac group compared with the control group, indicating that Ac_5_3F_ax_Neu5Ac injections potentiate the infiltration of immune cells into the tumor mass (Figure 6c). 

The effect of Ac_5_3F_ax_Neu5Ac on the immune cell composition, such as T-lymphocytes (CD4^+^ and CD8^+^), B-cells, Natural Killer (NK) cells, granulocytes, monocytes, and macrophages, revealed an increase in the percentage of infiltrating T cell population (CD3^+^) in treated-mice tumors compared with control-mice tumors, being significant for 10 mg/kg Ac_5_3F_ax_Neu5Ac-treated tumors. In particular, we detected a significant increase in the CD4^+^ and CD8^+^ T cell population in the 10 mg/kg Ac_5_3F_ax_Neu5Ac group with respect to control tumors (Figure 6d–f). This increase was also confirmed by IHC with anti-CD3, which showed higher numbers of T lymphocytes in the tumors of the treated groups, which are mostly located at the periphery of the tumors; moreover, Ac_5_3F_ax_Neu5Ac injections increased the percentage of NK cells, especially in the 10 mg/kg Ac_5_3F_ax_Neu5Ac group (Figure 6g). The percentage of myeloid regulatory cells (granulocytes and monocytes) in the 10 mg/kg group showed an increasing trend, while the percentage of B cells and macrophages remained unaltered in that group with respect to the control one (Figure 6h–k).

These results collectively suggest that Ac_5_3F_ax_Neu5Ac treatment at the dose of 10 mg/kg significantly altered the immune cell component of the tumor microenvironment by increasing the infiltrating T cell population, including T CD8^+^, and by an increasing trend of NK cells, altogether enhancing the immune response against the tumor. 

## 4. Discussion

The ST inhibitor, Ac_5_3F_ax_Neu5Ac, has been described to reduce SA determinants in human leukemia cells and in several murine melanoma cells, at doses that range from 64 µM to 300 µM [20]. In this study, we have described that Ac_5_3F_ax_Neu5Ac treatment at similar doses (100–400 µM) was effective in human and murine PDA cells and led to a generalized decrease of their cell sialome. 

In human PDA cells, Ac_5_3F_ax_Neu5Ac treatment resulted in a marked reduction of sLe^x^ expression (by 80–85%) on the cells that express sLe antigens, BxPC-3 and Capan-1, which is in line with the findings described by Natoni et al. [20], and led to a reduction of 55-85% in α2,3-SA levels, which is in accordance with the data showed by Rillahan and Büll [11,19]. The decrease in α2,6-SA was lower (28–38%) than for α2,3-SA (55–85%) for all human PDA cell lines, which is in accordance with Bull et al. [11] and suggests that Ac_5_3F_ax_Neu5Ac blocks α2,3-STs more effectively than α2,6-STs. Contrary to the trend observed in human PDA cells, in the murine KC cells, Ac_5_3F_ax_Neu5Ac treatment resulted in a higher decrease of α2,6-SA than α2,3-SA. Ac_5_3F_ax_Neu5Ac treatment caused a more efficiently and generalized reduction in SA levels than the one obtained by the KD of ST3GAL3 and ST3GAL4 in Capan-1 and BxPC-3 [15]. This could be explained by the action of Ac_5_3F_ax_Neu5Ac on the different α2,3-ST and α2,6-ST family members. Therefore, Ac_5_3F_ax_Neu5Ac could be considered as a promising candidate to reduce PDA cells hypersialylation. 

The high reduction in sLe^x^ and sLe^a^ observed in the Ac_5_3F_ax_Neu5Ac-treated BxPC-3 and Capan-1 cells can explain their impairment in binding to rhE-selectin (60–75%) in percentages that were in consonance with the reductions described for the corresponding PDA cells lines KD for ST3GAL3 and ST3GAL4, which also showed important decreases in sialyl-Lewis expression [15]. Similarly, Natoni et al. and Rillahan et al. demonstrated that a reduction of sLe^x^ and sLe^a^ caused by Ac_5_3F_ax_Neu5Ac treatment led to a significant decrease in the interaction between tumor cells and E-selectin in human leukaemia and melanoma cells [19,20]; moreover, Soyasaponin-I, which is another ST inhibitor, was described to block sialylation and inhibit tumor cell adhesion to endothelial monolayers [21].

The relevance of sialyl-Lewis antigens in the adhesion of tumor cells to E-selectin has also been shown by the invalidated expression of FUT3 in Capan-1 cells, which resulted in a reduction of sialyl-Lewis antigens and was associated to an inhibition of the E-selectin adhesion and a decrease of the metastatic power of the genetically modified cells [22]. Similarly, the overexpression of ST3GAL3 and ST3GAL4 in PDA cell lines, which catalyze the final steps of sialyl-Lewis antigens biosynthesis, was associated with an increased adhesion and metastatic capacity of PDA cells in vitro and a higher metastasis formation when injected into athymic nude mice [13,14].

Several studies have demonstrated that SA determinants modulate cell-ECM adhesion and migratory processes of various human cancer models, including pancreatic, gastric, and breast cancer [13,14,15,23,24,25,26,27]. In the present study, we have shown that the sialylation blockade caused by Ac_5_3F_ax_Neu5Ac treatment reduced the migratory (22–30%) and invasive capacities (12–25%) of the human and murine PDA cell lines, which is in agreement with the findings described by Büll et al. in melanoma cells [11]. Likewise, results obtained with other STs inhibitors (SsaI and AL10) showed that inhibition of α2,3-sialylation impaired cell motility, adhesion, and migration through ECM components and cell invasion in ovarian and lung cancer cells [21,28,29]. In the same line, human PDA cells with higher α2,3-SA levels correlated with a more migratory phenotype [30].

Furthermore, previous studies have stated that the upregulation or downregulation of ST3GAL3 and ST3GAL4, and the subsequent increase or decrease of sLe^x^ expression levels, respectively, results in increased or decreased migration and invasive phenotype, respectively, of either PDA cells [13,14,30] or other cancer models such as gastric or breast cancer [25,26]. In this work, we also performed blocking experiments by treating the human PDA cells with anti-sLe^x^ and anti-sLe^a^ antibodies. In accordance with previous results, we found that blocking sLe^x^ led to a significant reduction in BxPC-3 and Capan-1 cell migration and invasion abilities, which reinforces the importance of sLe^x^ antigen in the modulation of invasion and migration processes.

The administration of Ac_5_3F_ax_Neu5Ac to the mice was performed intratumorally to retain the inhibitor in the tumor mass and limit systemic exposure, since Macauley and colleagues had previously described that the intravenous administration of 300 mg/kg of inhibitor caused severe nephrotoxicity and ended in kidney failure [31]. We found that after two weeks of intratumoral administration, tumor growth was slowed down significantly in the 10 mg/kg group in comparison to control groups. Interestingly, and in in line with Büll et al., [12] we found that Ac_5_3F_ax_Neu5Ac had the potency to reduce SA expression in the established subcutaneous pancreatic tumors, with this decrease being significant in the reduction of α2,3-SA in the 10 mg/kg group; moreover, blocking SA expression altered the tumor microenvironment, leading to an increase of the immune cell to tumor cell ratio in the treated tumors. Although the infiltration of immune cells was low, which is in accordance with the fact that PDA tumors are described to have low immune component [32], we detected a marked increase in tumor infiltrating CD4^+^ and CD8^+^ T cells and NK cells, which are indicators of a reversion of the immunosuppressive tumor microenvironment [33,34]. Although it was not statistically significant, we also observed increasing percentages of granulocytes and monocytes, cells that are usually classified as immunosuppressive ones. Nonetheless, it has been described that myeloid cells can adopt a pro- or anti-tumorigenic profile depending on the tumor microenvironment composition and sialic acids expressed by tumor cells [35], thereby indicating that further studies are warranted to clarify the role of myeloid derived suppressor cells in PDA models [36]. Referring to mice survival, we could not significantly determine whether the administration of the inhibitor and the concomitant reduction of tumour growth could prolong the mice survival time. In that sense, some methodological considerations could be addressed and improved for future experiments. First, in terms of SA expression and tumour generation, we selected the Ac_5_3F_ax_Neu5Ac sensitive murine pancreatic cancer cell line, KC; however, we found that in general the tumours grew faster than expected and wounds appeared in some of them. Second, considering the total body weight of the mice, we hypothesize that the start of inhibitor treatment could be established at a smaller tumour volume, so we could extend the tumour follow-up. Third, it would be of special interest to generate orthotopic tumours in the pancreas as they offer tissue-site-specific pathology, which is a more realistic influence of the tumour microenvironment and allows the study of Ac_5_3F_ax_Neu5Ac’s effect on metastasis formation.

The molecular mechanisms by which SA blockade leads to these changes in the tumor microenvironment remains undetermined. However, there is growing evidence that blocking SA interactions of tumor cells with immune modulatory Siglecs of immune cells hampers the formation of an immunosuppressive tumor microenvironment [37,38,39,40]. Recently, Rodriguez et al. revealed that enhanced α2,3-sialylation of PDA cells is sensed by Siglec-7 and Siglec-9 and promotes the differentiation of monocytes into macrophages with an immune-suppressive phenotype [18]. Siglec-7 and Siglec-9 can bind to sialic acids on both *O*- and *N*-glycans, such as sialyl-T or sLe^x^ generated by the activity of two enzymes, ST3Gal1 and ST3Gal4, which are upregulated in PDA patients [18,41]. Perdicchio et al. described that the B16 melanoma tumors with low SA content grew slower in vivo than the corresponding hypersialylated tumors and altered the Treg/Teffector balance, thereby favoring immunological tumor control dependent on an increased influx and activity of NK cells [42].

The sialic acid blockade has an extensive effect on tumor cells and the tumor microenvironment. It has been postulated that the increased cell death and growth inhibition in melanoma tumors after Ac_5_3F_ax_Neu5Ac treatment can result from the higher number and activation status of cytotoxic T cells [12], which could partially explain the observed reduction in tumor growth of the 10 mg/kg treated group. Büll et al. observed increased clustering between desialylated melanoma cells and CD8^+^ T cells with a transgenic T-cell receptor compared to control cells [12], suggesting that the SA blockade facilitates tumor cell–T cell interactions. All these findings, in accordance with our results, support the idea that the SA blockade potentiates the killing of tumor cells by cytotoxic T-cells.

## 5. Conclusions

To our knowledge, this work is the first that reports the effect of the ST inhibitor Ac_5_3F_ax_Neu5Ac in PDA cells and animal models. We have shown that the reduction of PDA cell sialylation can revert the invasive phenotype, and reduce tumor growth and the immunosuppressive microenvironment in vivo. Further studies are warranted to understand the underlying mechanisms, but the data presented in this work might pave the way for using these metabolic inhibitors to evaluate the role of sialylated glycans in tumor progression and for their possible therapeutic application.

## Figures and Tables

**Table 1:** Lectins and antibodies for Flow cytometry (FC), spectral FC, WB, and IHC.

Antibody/Biotinylated Lectin /Conjugated Reagent	Clone or Host/Isotype	Dilution	Use	Supplier
anti-sLe^x^	CSLEX	1/10 (FC)	FC/WB	Cat# 563529, BD Biosciences (San Jose, CA, USA)
		1/70 (WB)		
anti-sLe^a^	121SLE	1/500 (FC)	FC/WB	Cat# ab3982, Abcam (Cambridge, UK)
		1/1000 (WB)		
anti-Tubulin	B-7	1/500	WB	Cat# sc-5286, Santa Cruz Biotechnology (Dallas, TX, USA)
Biotinylated SNA		1/100 (FC)	FC/WB	Cat# B-1305, Vector Laboratories (Burlingame, CA, USA)
		1/1000 (WB)		
Biotinylated MAA-II		1/50 (FC)	FC/WB	Cat# B-1265, Vector Laboratories
		1/500 (WB)		
Biotinylated PNA		1/100	FC	Cat# B-1075-5, Vector Laboratories
Peroxidase-Conjugated goat anti-Mouse IgG + IgM		1/4000	WB	Cat#115-035-06, Jackson immune Research (West Grove, PA, USA)
Peroxidase-Conjugate goat anti-Mouse IgG		1/1000	WB	Cat# 401215, Millipore (Darmstadt, Germany)
Streptavidin-HRP Conjugate		1/100,000	WB	Cat# GERPN1231, GE Healthcare (Little Chalfont, UK)
Anti-mouse IgG conjugated to Alexa Fluor 488		1/400	FC	Cat# A-11029, Thermo Fisher Scientific (Waltham, MA, USA)
Streptavidin conjugated to Alexa Fluor 488		1/1000	FC	Cat# S32354, Invitrogen (Carlsbad, CA, USA)
anti-phosphoHistone H3	MC463	1/300	IHC	Cat# 04-817, Millipore
anti-CD3		1/100	IHC	Cat# ab5690, Abcam
horse biotinylated antibody anti-IgGs		1/100	IHC	Cat# BA-1400, Vector Laboratories
avidin-peroxidase complex			IHC	Cat# BA-1400, Vector Laboratories
Anti-mouse CD16 antibody	Rat IgG2a, λ	1/100	Spectral FC	Cat# 101302, Biolegend (San Diego, CA, USA)
Brilliant Violet 510™ anti-mouse CD8a Antibody	Rat IgG2a, κ	1/1000	Spectral FC	Cat# 100751, BioLegend
APC anti-mouse CD3 Antibody)	Rat IgG2b, κ	1/500	Spectral FC	Cat# 100235, BioLegend
PerCP-Cy™5.5 Rat Anti-Mouse CD45	Rat LOU	1/200	Spectral FC	Cat# 567310, BD Biosciences
APC-Cyanine7 anti-mouse CD4	Rat DA, also known as DA/HA IgG2a, κ	1/400	Spectral FC	Cat# 552051, BD Biosciences
PE/Cyanine7 anti-mouse Ly-6G Antibody	Rat IgG2a, κ	1/1000	Spectral FC	Cat# 127617, BioLegend
FITC anti-mouse/human CD11b Antibody	Rat IgG2b, κ	1/4000	Spectral FC	Cat# 101205, BioLegend
PE-Cy™5 Anti-Mouse CD45R/B220	Rat IgG2b, κ	1/200	Spectral FC	Cat# 561879, BD Biosciences
Brilliant Violet 785™ anti-mouse F4/80 Antibody	Rat IgG2a, κ	1/100	Spectral FC	Cat# 123141, BioLegend
Alexa Fluor^®^ 700 anti-mouse Ly-6C Antibody	Rat IgG2c, κ	1/200	Spectral FC	Cat# 128023, BioLegend
PE/Dazzle™ 594 anti-mouse CD49b (pan-NK1.1 cells) Antibody	Rat IgM, κ	1/1000	Spectral FC	Cat# 108923, BioLegend
Brilliant Violet 650™ anti-mouse/human CD45R/B220 Antibody	Rat IgG2a, κ	1/100	Spectral FC	Cat# 103241, BioLegend

## Data Availability

All data generated or analyzed in this study are included in this published article and Supplementary Information.

## Supplementary Materials

The following supporting information can be downloaded at: https://www.mdpi.com/article/10.3390/cancers14246133/s1, Figure S1: Analysis of Ac_5_3F_ax_Neu5Ac effect on cell sialylation in BxPC-3, Capan-1 and Panc-1 cells by WB. File S1: Full Western blot images.
