# Supplementary material for: Sialyltransferase Inhibitor Ac53FaxNeu5Ac Reverts the Malignant Phenotype of Pancreatic Cancer Cells, and Reduces Tumor Volume and Favors T-Cell Infiltrates in Mice
Source: Cancers (Basel). 2022 Dec 12;14(24):6133. doi: 10.3390/cancers14246133 (PMC9776040; doi:10.3390/cancers14246133)
Supplement: Supplementary file 1 [file cancers-14-06133-s001.zip › cancers-2053012-supplementary/cancers-2053012-Supplementary Figure S1.pdf]

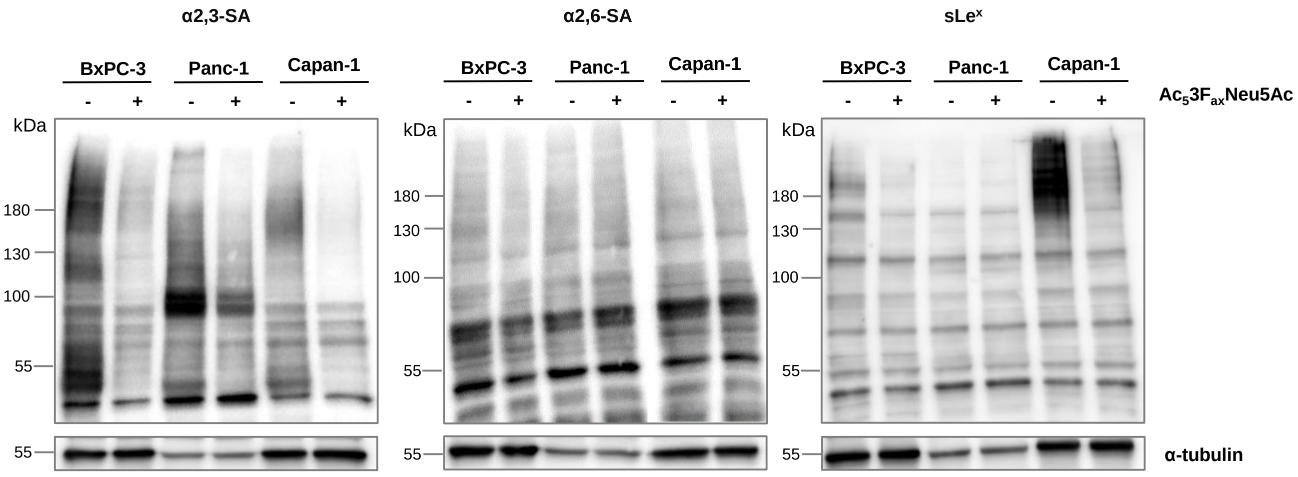

Supplementary Figure S1. Analysis of Ac$_5$3F$_{ax}$Neu5Ac effect on cell sialylation in BxPC-3, Capan-1 and Panc-1 cells by WB. Representative blots of the expression of the sialylated glycan structures α2,3-SA, 2,6-SA and sLe^x from the cell lysates of treated and control BxPC-3, Capan-1 and Panc-1 cells (top). Immunodetection of tubulin after stripping of the membranes, as loading control (bottom).
