# Supplementary figures and images for: Sialyltransferase Inhibitor Ac53FaxNeu5Ac Reverts the Malignant Phenotype of Pancreatic Cancer Cells, and Reduces Tumor Volume and Favors T-Cell Infiltrates in Mice
Source: Cancers (Basel). 2022 Dec 12;14(24):6133. doi: 10.3390/cancers14246133 (PMC9776040; doi:10.3390/cancers14246133)

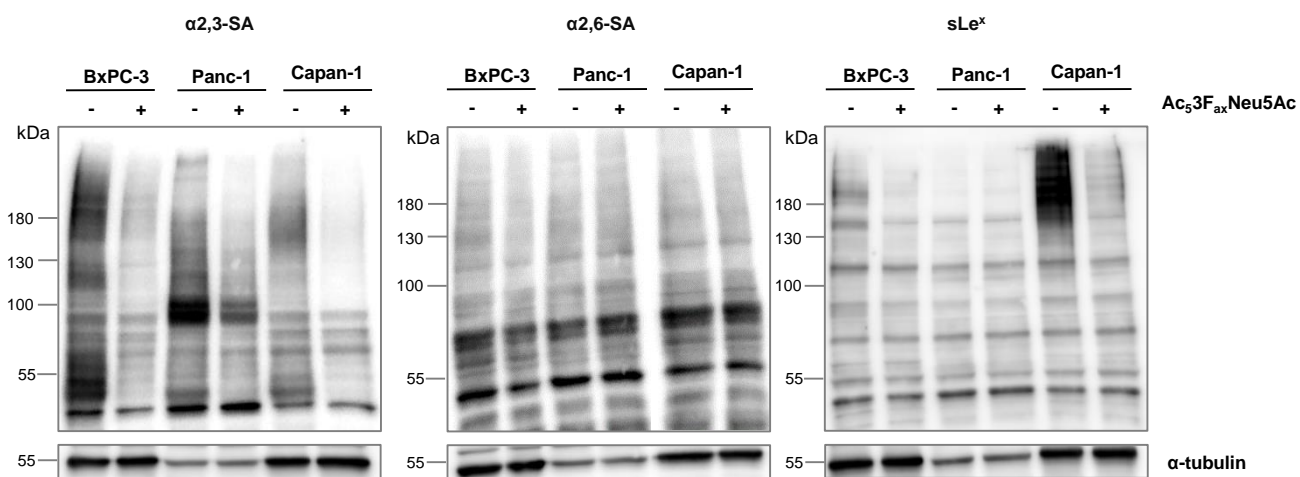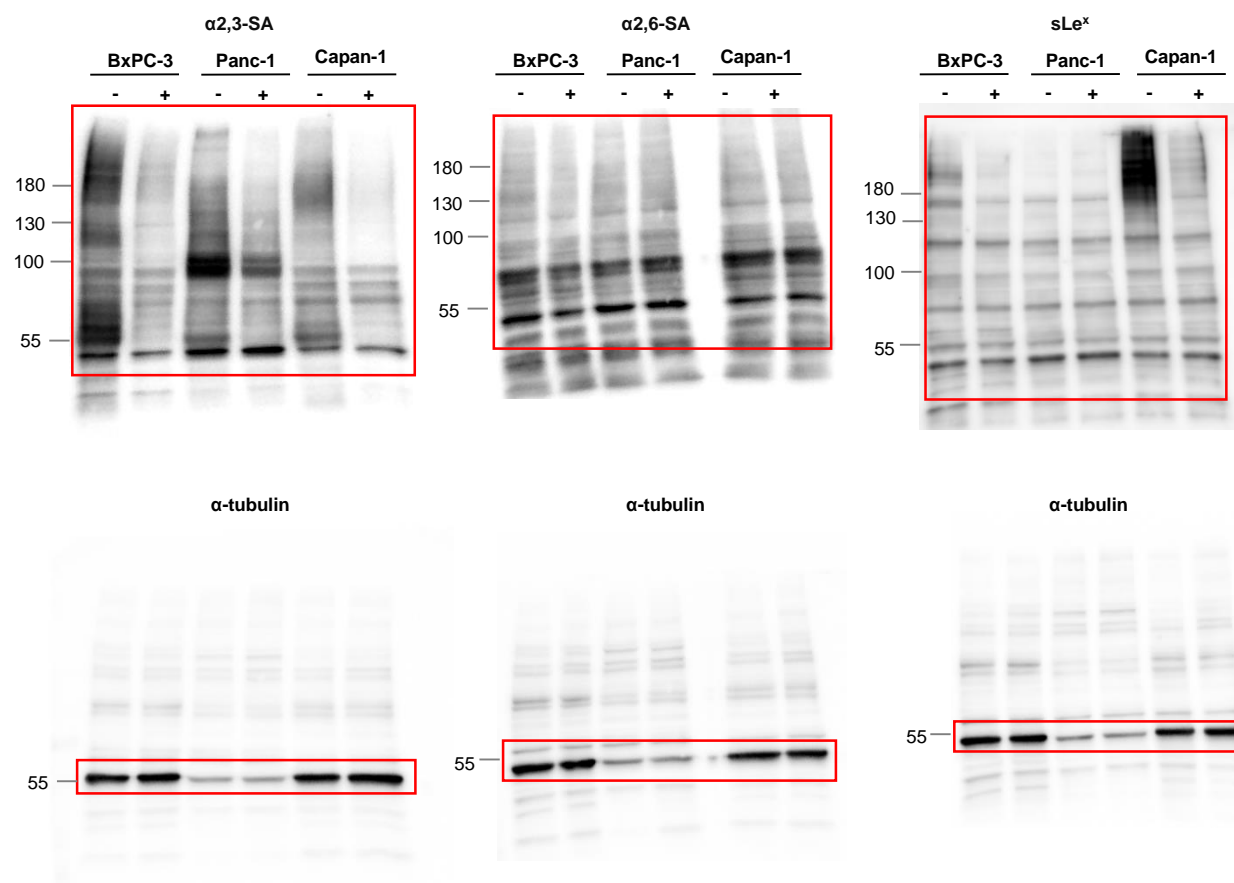

Supplement: Supplementary file 1 [file cancers-14-06133-s001.zip › cancers-2053012-supplementary/cancers-2053012-File S1.pdf]
